# Integrated analysis of transcriptome and proteome changes related to the Ogura cytoplasmic male sterility in cabbage

**DOI:** 10.1371/journal.pone.0193462

**Published:** 2018-03-12

**Authors:** Miaomiao Xing, Chao Sun, Hailong Li, Shilin Hu, Lei Lei, Jungen Kang

**Affiliations:** Beijing Vegetable Research Center, Beijing Academy of Agriculture and Forestry Sciences, Key Laboratory of Biology and Genetic Improvement of Horticultural Crops (North China), Ministry of Agriculture, Beijing, China; Huazhong University of Science and Technology, CHINA

## Abstract

Cabbage (*Brassica oleracea* L. var. *capitata*), an important vegetable crop in the Brassicaceae family, is economically important worldwide. In the process of hybrid seed production, Ogura cytoplasmic male sterility (OguCMS), controlled by the mitochondrial gene *orf138*, has been extensively used for cabbage hybrid production with complete and stable male sterility. To identify the critical genes and pathways involved in the sterility and to better understand the underlying molecular mechanisms, the anther of OguCMS line R2P2CMS and the fertile line R2P2 were used for RNA-seq and iTRAQ (Isobaric Tags for Relative and Absolute Quantitation) proteome analysis. RNA-seq analysis generated 13,037,109 to 13,066,594 SE50-clean reads, from the sterile and fertile lines, which were assembled into 36,890 unigenes. Among them, 1,323 differentially expressed genes (DEGs) were identified, consisting of 307 up- and 1016 down-regulated genes. For ITRAQ analysis, a total of 7,147 unique proteins were identified, and 833 were differentially expressed including 538 up- and 295 down-regulated proteins. These were mainly annotated to the ribosome, spliceosome and mRNA surveillance pathways. Combined transcriptomic and proteomic analyses identified 22 and 70 genes with the same and opposite expression profiles, respectively. Using KEGG analysis of DEGs, gibberellin mediated signaling pathways regulating tapetum programmed cell death and four different pathways involved in sporopollenin synthesis were identified. Secretion and translocation of the sporopollenin precursors were identified, and the key genes participating in these pathways were all significantly down-regulated in R2P2CMS. Light and transmission electron (TE) microscopy revealed fat abnormal tapetum rather than vacuolization and degradation at the tetrad and microspore stages of the OguCMS line. This resulted in the failed deposition of sporopollenin on the pollen resulting in sterility. This study provides a comprehensive understanding of the mechanism underlying OguCMS in cabbage.

## Introduction

Cabbage (*Brassica oleracea* var. *capitata*) is the most widely grown vegetable crop in the world. Of the leafy vegetables, cabbage production is second only to Chinese cabbage (*Brassica rapa*) in China. Currently, the Ogura cytoplasmic male sterility (OguCMS) system, which is controlled by the mitochondrial gene *orf138*, is becoming the most common method of cabbage F1 hybrid production [1]. As cytoplasmic male sterile lines and maintainer lines have the same nuclear genome, OguCMS is not only an important tool for genetic improvement and utilization of heterosis in crops, but also a classic model to explore cytoplasm mechanism and reverse signaling pathways in pollen development biology [2,3]

Mitochondrial mutations can lead to male infertility. Previous studies have focused on screening and isolating mitochondrial genes associated with sterility. A recent study showed that the mitochondrial genome of normal *Brassica* plants contain 33 protein-coding genes and 3 rRNA genes and non-syntenic to the mitochondrial genome of maintainer lines. In OguCMS, there is a unique high rearrangement region (15255 bp) with *orf138* located at its edge and near to *trnfM* and *atp9* [4]. Despite the non-homologous sequence between *orf138* and other CMS genes, the genes share common traits. They derive from high rearrangement of the mitochondrial genome, are co-transcribed with ATPase, and encode a small protein with hydrophobic areas. How the mitochondrial sterile genes, including *orf138*, retrograde regulate nuclear genes remains unclear. The CMS system may result in changes to the respiratory chain complex and decreased activity of ATP synthase and photosynthetic pigment oxidase [5–9]. In the sunflower CMS line, orf522 competes with ATP8 resulting in decreased stability and synthetic efficiency of ATPase by binding with an ATP synthase subunit [7]. The same abortive mechanism of ATP6-orfH79 appeared in rice HonglianCMS [9]. While for *orf138* in OguCMS, the spontaneous promotion of the absence of fertility occurred instead of combining to the respiratory complex.

Only recently has research into CMS modulated by mitochondrial retrograde regulation (MRR) in higher plants been published. Rhoads et al [10] provided an overview of MRR in plants and Yang et al [11] hypothesized that the mechanism of CMS was modulated by MRR. Fujii et al [12,13] inferred that in rice CW-type CMS, the Ca^2+^ signaling pathway may participate in sterility development regulated by MRR and dysfunction of the mitochondrial phosphatase gene *DCW11* which showed similar abortion characteristics. Transcriptome and proteome analysis identified genes and pathways involved in CMS lines. Microarray analysis of *Raphanus sativus* OguCMS indicated that genes involved in flavonoid biosynthesis were inhibited and the key enzyme, chalcone synthase (CHS), was particularly inhibited [14]. Microarray analysis of Chinese cabbage OguCMS showed that genes related to pollen development, auxin and stress response and ATP synthesis had delayed expression leading to dysfunctional pollen [15]. Identification of differentially expressed proteins by 2-DE gel in a wheat CMS line showed that their sterility is related to active oxygen accumulation, energy metabolism perturbation, the pentose phosphate pathway, programmed cell death, and glycolysis [16]. Transcriptome analysis of DEGs related to OguCMS in cabbage identified differentially expressed genes associated with energy and carbohydrate metabolism, the Ca^2+^ signaling pathway, transcription factors and other genes such as HSPs and STPs [17]. To investigate MRR by *orf138*, we previously compared gene expression profiles of the anthers between OguCMS and the maintainer line using genome-wide microarray of *Arabidopsis*. There were 71 differentially expressed genes in the OguCMS anther located on the mitochondria and a series of functional genes associated with anther development such as transcription factors: *BoBHLH1*, *BoMYB1*, *BoAHL16*. Functional analysis of these TFs was performed by yeast two hybrid or transgenic technology, confirming their role in regulating the abortion pathways in OguCMS [18–21].

The aim of this study was to elucidate the mechanism of MRR by *orf138* in OguCMS by integrating transcriptome and proteome data.

## Material and methods

### Material

**Fig 1:**
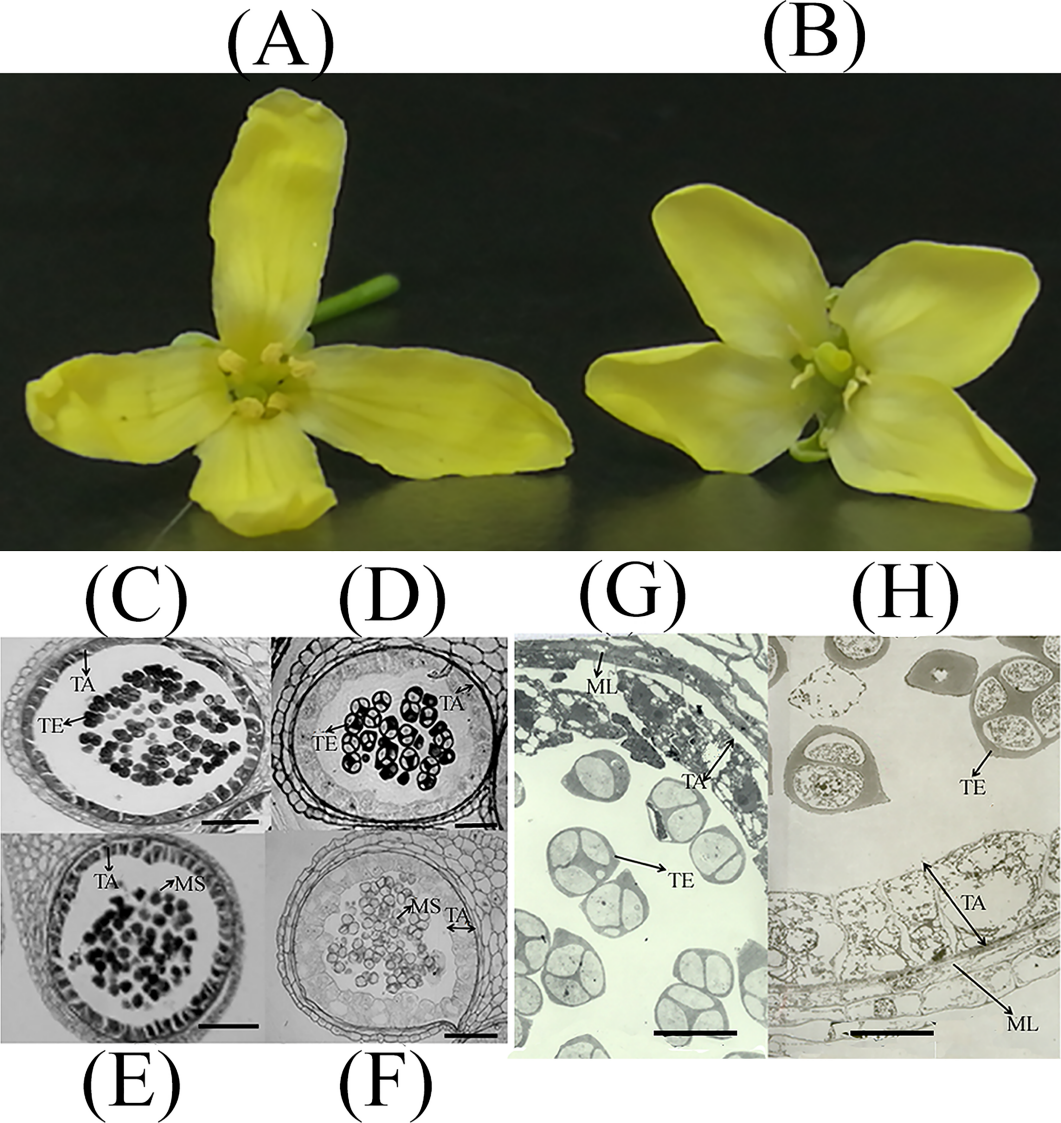
Flowers and Light and transmission electron (TE) micrographs of the tetrad (anther development stage: 7–8) and uninucleate microspore stage (anther development stage: 9–10) in R2P2 (C, D, G) and R2P2CMS (E, F, H). **(A)**: Flowers of R2P2; **(B)**: Flowers of R2P2CMS. **(C), (D):** Optical micrographs of anthers at the tetrad stage. **(E), (F):** Optical micrographs of anthers at the uninucleate microspore stage *Scale bar* = 50 μm. **(G), (H):** TE micrographs of anthers at the tetrad stage. TA: tapetum. TE: tetrad. MS: microspore. ML: middle layer. *Scale bar* = 20 μm.

R2P2CMS, a BC8 CMS cabbage (*B*. *oleracea var*. *capitata*) line, and its maintainer line R2P2 were grown under the same conditions. After flowering, six inflorescence from individual plants were sampled from each line. The inflorescence were randomly placed into two groups of three giving two replicates per line, namely R2P2CMS-1, R2P2CMS-2, R2P2-1, R2P2-2 for both RNA-seq and ITRAQ quantification. The anthers were removed and all four samples were immediately frozen in liquid nitrogen then stored at -80°C for total RNA and protein extraction. Flowers of R2P2CMS and R2P2 are showed in Fig 1.

### Light and transmission electron microscopy

Light microscopy was carried out according to Kang et al [22]. Buds of R2P2CMS and R2P2 at different stages were collected and fixed overnight in FAA, dehydrated in gradient ethanol, embedded in Spurr’s epoxy resin and sectioned into 1 μm thick slices. Sections were then stained (1% toluidine blue, 42°C, 1–2 h) and observed under the microscope.

Transmission electron (TE) microscopy was carried out according to Kang, et al [23]. Buds of different sizes were fixed overnight in 4% glutaraldehyde, rinsed overnight with 200 mM phosphate buffer (pH 7.0), post-fixed (1% osmium tetroxide, 2 h) and dehydrated in an ethanol series (30 min at each concentration). The buds were then embedded in Spurr’s epoxy resin (60°C, 3 d) and cut into 60–90 nm sections. Sections were then stained (4% uranyl acetate, 20 min and lead citrate, 3 min) and observed using TEM (H-8100, Hitachi).

### RNA-Seq: Sample preparation and data analysis

Total RNA was isolated using the RNAprep Pure Plant Kit (TIANGEN) according to the manufacturer’s protocol.

Differential expression analysis was performed using Noiseq algorithm [24] with a cutoff of probability ≥0.8 &│log2Ratio(R2P2CMS/R2P2)│≥1. WEGO software [25] was used for GO analysis. KEGG is used to perform pathway analysis. Pathways with Q-value ≤ 0.05 were considered as significantly enriched pathways.

### Validation of differentially expressed genes (DEGs) by qRT-PCR

Identified DEGs were validated using qRT-PCR. cDNA were synthesized from the same samples used for the high-throughput sequencing. *GAPDH* was used as an internal reference. qRT-PCR was performed using SYBR Green 1 (TIANGEN) on a Light Cycler 480 II Real-Time PCR Detection System (Bio-Rad, USA). The reaction was carried out in a total volume of 20 μL containing 2× SuperReal PreMix Plus (10 μL), diluted cDNA mix (2 μL), each primer (10 mM, 0.6 μL) and RNase-free ddH_2_O (6.8 μL). The amplification program was as follows: (1) 95°C for 15 min; (2) 40 cycles of 95°C for 10 s, 60°C for 20 s and 72°C for 20 s; (3) melting curve: 95°C for 5 s, 65°C for 60 s, 97°C (continuous); (4) 40°C for 30 s. Relative expression was calculated using 2^-ΔΔCt^ method. Genes and primer sequences are showed in S1 Table.

### ITRAQ analysis

#### Protein extraction

Tissue was ground into a powder in liquid nitrogen and 5 ml of lysis buffer was added. PMSF and EDTA were added to achieve final concentrations of 1 mM and 2 mM, respectively. After 5 mins, DTT was added to a final concentration of 10 mM. The suspension was sonicated for 15 minutes and then centrifuged at 25,000 ×g for 20 mins. The disulfide bond of the supernatant was reduced with 10 mM DTT at 56°C for 1 h. To block the cysteine, 55 mM IAM was added and incubated in the dark for 45 mins. Five-fold chilled acetone was added to the supernatant for 2 h at -20°C. After centrifugation at 25,000 ×g for 20 mins, the supernatant was discarded and the pellet dried in air for 5 mins. The dried pellet was dissolved in 200 μl 0.5 M TEAB and sonicated for 15 mins. Finally, the solution was centrifuged at 25,000 ×g for 20 mins.

The Bradford method was used to measure the concentration of the protein. Protein solutions (100 μg) from each sample were digested with trypsin (protein: trypsin = 20:1) at 37°C, first for 4 h and then for 8 h.

#### iTRAQ labeling and SCX chromatography

After digestion, peptides were vacuum centrifuged until dryness and redissolved in 0.5 M TEAB. iTRAQ labeling was performed using iTRAQ reagent 4-plex kit(AB Sciex Pte. Ltd) based on the manufacturer's protocol: allow iTRAQ® Reagents– 4 plex to reach room temperature, to each iTRAQ Reagent– 4 plex, add 50 μL of isopropanol; Vortex and then spin. transfer the contents of one iTRAQ Reagent– 4 plex vial to one sample tube; Vortex to mix, spin; If pH is <7.5, add up to 5 μL Dissolution Buffer, then incubate at room temperature for 2 hours, then the labeled peptides were obtained. Our samples were labeled as R2P2_1_113, R2P2_1_119, R2P2CMS_1_114 and R2P2CMS_2_121. Labeled peptides were then fractionated using strong cation-exchange (SCX), SCX chromatography was performed using a Shimadzu LC-20AB HPLC Pump system.

#### LC-ESI-MS/MS analysis based on Q exactive

Fractions were suspended using buffer A (2% ACN, 0.1% FA) and centrifuged at 20,000 xg (10 min). A 10 μL aliquot of supernatant at a final concentration of 0.5 μg/μL was loaded onto a 2 cm C18 trap column and LC-20AD nano-HPLC. Peptides were eluted onto a 10 cm analytical C18 column packed in-house. Samples were loaded at a speed of 8 μL/min for 4 min, then run at 300 nL/min within a 41 min gradient from 5% to 35% B (98% ACN, 0.1% FA), followed by a 5 min linear gradient to 80% B and maintained for 4 min.

Peptides were subjected to nanoelectrospray ionization followed by MS/MS in a Q EXACTIVE coupled online to the HPLC. Intact peptides were detected using an Orbitrap at a resolution of 70,000. Peptides were selected for MS/MS using HCD operating mode with a normalized collision energy setting of 27.0. The resolution was 17,500 for detection of ion fragments. A data dependent-procedure that alternated between one MS scan followed by 15 MS/MS scans was applied for the 15 most abundant precursor ions above a threshold ion count of 20,000 (MS) with a Dynamic Exclusion duration of 15 s. The electrospray voltage was 1.6 kV. Automatic gain control was used to optimize the spectra and its target was 3e6 for full MS and 1e5 for MS 2. The m/z scan range was 350~2000 Da for MS scans and 100~1800 Da for MS 2 scans.

Protein identification was analyzed with the cutoff: Mascot Percolator [26] Q-value < = 0.01 and protein quantification was applied using BGI's IQuant [27]. Proteins with a 1.2 fold change and a Q-value of less than 0.05 were determined as differentially expressed proteins (DEPs).

## Results

### Cytological analysis of anthers from R2P2 and R2P2CMS

As with our previous cytological analysis of the anthers of OguCMS cabbage [22], the abnormal pollen development of the OguCMS cabbage line was mainly due to the proliferation and expansion of the tapetum at the anther development stage 9–12 according to Sanders et al [28]. and tapetum PCD from stage 5 to the tetrad stage. To further investigate the tapetum development in anthers of OguCMS cabbage, we performed light and transmission electron microscopy. Paraffin sections (Fig 1) revealed enlarged abnormal tapetum at the tetrad stage of the OguCMS line resulting in delayed deposition of sporopollenin. Compared to R2P2 (Fig 1C and 1D), light microscopy of the tetrads (anther development stage:7–8) and the uninucleate stage (anther development stage:9–10) revealed a fat abnormal tapetum in R2P2CMS (Fig 1E and 1F). Furthermore, the hypertrophic abnormal tapetum at the tetrad stage was observed in R2P2CMS (Fig 1H) using transmission electron (TE) microscopy.

### Correlation between samples

Correlation analysis of gene expression level among samples was used to verify experimental reliability and sampling accuracy. The correlation value between the two replicates was calculated based on FPKM, and should be ≥0.92 as per the Encode plan. Correlation between R2P2CMS-1 and R2P2CMS-2 was 0.9479 and 0.9341 between R2P2-1 and R2P2-2 (Table 1). High correlation suggests the replicate samples have high reliability and repeatability, ensuring subsequent analysis reflects real differences in gene expression between R2P2CMS and R2P2.

**Table 1:** Correlation between samples.

Sample	R2P2CMS-2	R2P2-2	R2P2CMS-1	R2P2-1
R2P2CMS-2	1	0.7220845	0.9478632	0.5330607
R2P2-2	0.7220845	1	0.6504894	0.9341372
R2P2CMS-1	0.9478632	0.6504894	1	0.5008382
R2P2-1	0.5330607	0.9341372	0.5008382	1

### Differentially expressed genes in R2P2CMS and R2P2 by RNA-seq analysis

RNA-seq analysis generated 13,037,109 to 13,066,594 SE50-clean reads, which were assembled into 36,890 unigenes. A total of 1323 genes (307 up- and 1016 down-regulated genes) with probability ≥0.8 &│log2Ratio(R2P2CMS/R2P20029│≥1 were differentially regulated in R2P2CMS. DEGs were mainly assigned (GO analysis) into three groups: (1) response to stimulus including light intensity and reactive oxygen species; (2) cell wall organization or biogenesis; (3) pollen development. There were 22 DEGs assigned to pollen development, pollen wall assembly and pollen exine formation, and all were down-regulated in R2P2CMS as showed in Table 2.

**Table 2:** Twenty-two DEGs functionally assigned to pollen development.

GO (biological process)			GeneID	Gene length (bp)	Means-R2P2	Means-R2P2CMS	log2Ratio (R2P2CMS/R2P2)	Probability	Description
Pollen development			Bol019328	5208	13.31	0.19	-6.1683	0.8519	AT2G13680.1 CALS5 (callose synthase 5)
			Bol028325	1428	300.57	18.09	-4.0544	0.9021	AT4G20050.1 Pectin lyase-like superfamily protein
	Pollen wall assembly		Bol034656	1179	82.68	9.51	-3.1199	0.8779	AT4G34850.1 LAP5 (Chalcone and stilbene synthase family protein)
			Bol013698	1179	99.67	18.93	-2.3969	0.8691	
			Bol025267	1182	229.35	63.05	-1.8631	0.8557	AT2G43340.1 LAP6(Chalcone and stilbene synthase family protein)
			Bol037639	1245	355.51	105.465	-1.7531	0.8538	AT3G59530.1 LAP3 | Calcium-dependent phosphotriesterase superfamily protein
			Bol045568	1242	57.39	11.63	-2.3028	0.8534	
			Bol010336	1530	127.51	40.1	-1.6689	0.8415	AT3G11980.1 MS2, FAR2 | Jojoba acyl CoA reductase-related male sterility protein
			Bol007277	1851	169.07	52.75	-1.6803	0.8458	
			Bol006196	1449	143.56	38.77	-1.8888	0.8536	AT4G14080.1 MEE48 | O-Glycosyl hydrolases family 17 protein
			Bol009974	1443	445.74	182.15	-1.2911	0.8155	
			Bol027052	1512	89.83	26.80	-1.7452	0.8396	
			Bol029814	1440	70.27	1.61	-5.4477	0.9216	AT2G19070.1 SHT | spermidine hydroxycinnamoyl transferase
			Bol042986	1005	34.64	0.01	-11.7580	0.9637	AT3G28840.1 Protein of unknown function
			Bol001202	2499	34.11	1.92	-4.1510	0.8671	AT3G03780.1 MS2
			Bol012512	1143	564.53	0.29	-10.9268	0.9991	AT2G19070.1 SHT | spermidine hydroxycinnamoyl transferase
			Bol042984	1941	37.56	0.06	-9.2898	0.9656	AT3G28780.1 Protein of unknown function
		Pollen exine formation	Bol018458	1260	54.20	2.355	-4.5244	0.8923	AT1G01280.1 CYP703A2
			Bol040704	1548	231.01	75.46	-1.6143	0.8404	
			Bol029711	4632	29.25	5.57	-2.3937	0.8272	AT1G62940.1 ACOS5
			Bol016365	954	181.92	57.39	-1.6646	0.8450	AT4G35420.1 DRL1
			Bol023932	1560	140.06	39.34	-1.8321	0.8501	AT1G69500.1 CYP704B1

### Validation of RNA-seq-based DEGs by qRT-PCR

**Fig 2:**
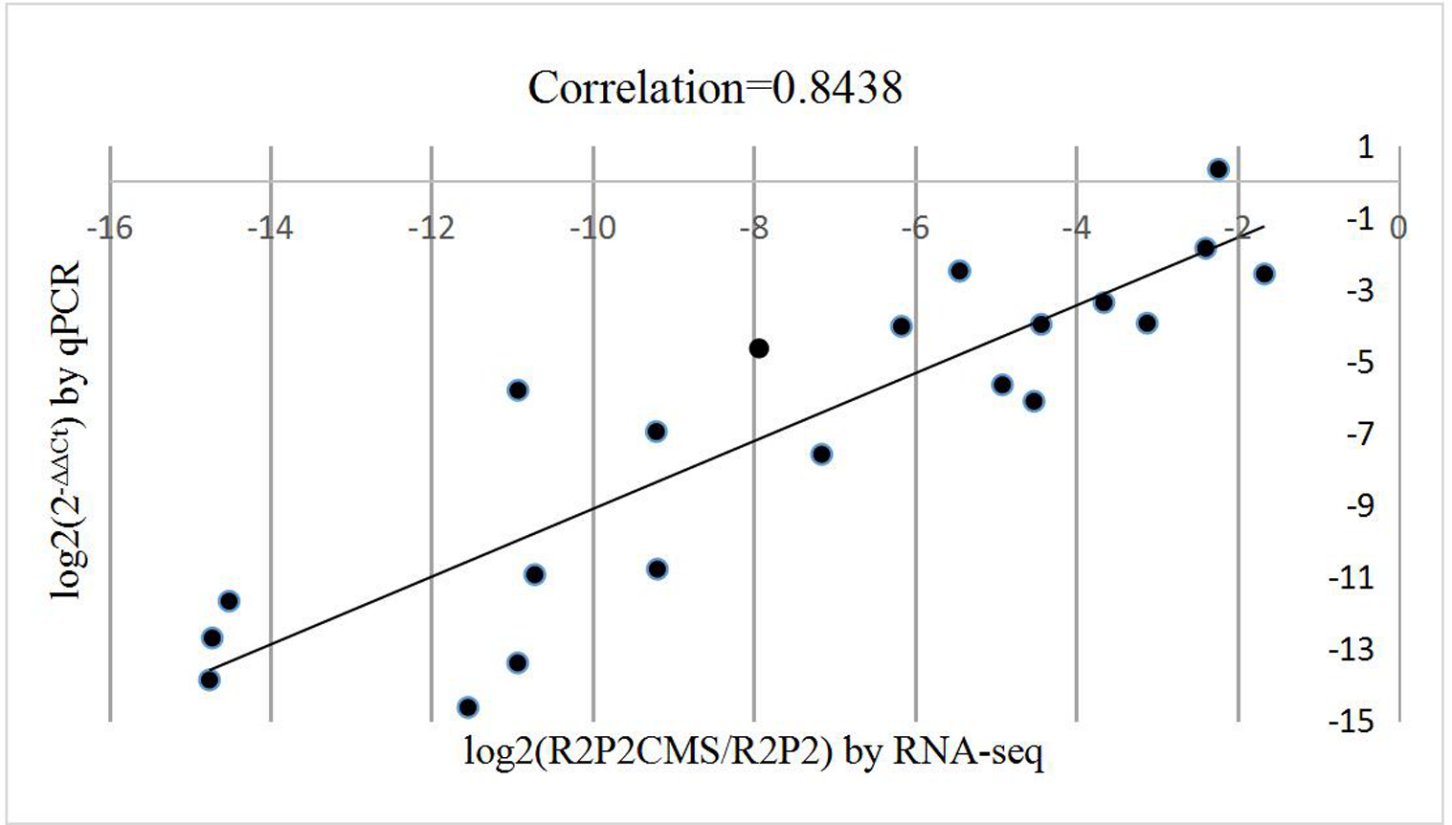
Correlation between RNA-seq and qRT-PCR for selected genes.

There were 23 DEGs selected for validation using qRT-PCR. Correlation analysis was performed to evaluate the two platforms. The RNA-seq data (log2 (R2P2CMS/R2P2)) and qRT-PCR data (log2(2^-ΔΔCt^)) for each DEG (S1 Table) was positively correlated at 0.8438 (Fig 2, correlation is significant at the 0.01 level).

### Factors related to tapetum programmed cell death (PCD)

Cytological analysis showed that proliferation and expansion of the tapetum appeared at the anther developmental stage 9–12. Furthermore, most of the microspores were compressed by abnormal hypertrophic tapetum cells in R2P2CMS. Two DEGs were identified (Bol017269, log2Ratio: -12.4592; Bol008912:-9.2109) encoding GA-regulated family protein[29,30], *MYB101* (Bol013459:-7.9363)[31–33] involved in GA mediated signaling pathway- triggered PCD, *TDF1/MYB 35* (Bol042967:-6.6075)[34], *PLP4* (Bol028944:-6.7823)[35] and *AMS* genes (Bol042692:-2.6678; Bol004758:-3.0183)[36,37], and all these genes were significantly down-regulated in R2P2CMS (S2 Table). Four DEGs were identified as belonging to the cysteine proteinase superfamily[38,39] (Bol041049:-14.5119, Bol015354:-2.0250, Bol041861:-7.6149, Bol023341:-11.0642) and were rarely expressed in R2P2CMS. Expression levels of *MYB101* (Bol013459), GA-regulated family protein (Bol008912) and cysteine proteinase (Bol041049) were verified by qRT-PCR with a value of log2(2^-PCR^ -4.6459, -6.9604, -11.6789.

### Essential biosynthesis pathways and key related genes in exine development

From our pathway enrichment results, sporopollenin biosynthesis such as fatty acid elongation (S3 Table), biosynthesis of unsaturated fatty acid (S4 Table), phenylpropanoid biosynthesis pathway (S5 Table), flavonoid biosynthesis (S6 Table), carotenoid biosynthesis (S7 Table), pathway of cutin, suberine and wax biosynthesis (S8 Table) and protein processing in ER (S9 Table) were among the top 20 significantly enriched pathways (pathways with Q-value ≤ 0.05).

All 8 DEGs identified as being involved in fatty acid elongation and 8 DEGs identified as being involved in biosynthesis of unsaturated fatty acid were down-regulated in R2P2CMS. Most of the genes involved in fatty acid elongation were KCS (3-ketoacyl-CoA synthase) genes.

All 24 DEGs (except Bol017968) involved in the phenylpropanoid biosynthesis pathway and 20 DEGs involved in flavonoid biosynthesis were down-regulated in R2P2CMS. Key genes participated both in flavonoid biosynthesis and phenylpropanoid biosynthesis or metabolic process by KEGG annotation included *DRL1* (Bol016365: -1.6646), *LAP5* (Bol013698: -2.3969; Bol034656: -3.1199), *LAP6* (Bol025267: -1.8631), *CYP78A7* (Bol043713: -5.2576) and *CYP98A8* (Bol039941: -7.1587). Other key phenylpropanoid biosynthesis genes included *ACOS5* (Bol029711: -2.3937) and *PAL1* (Bol037689: -1.5571; Bol025522: -2.1679) which were also identified as participating in gametophyte development annotated by GO. Other key DEGs involved in the flavonoid biosynthesis included *CYP703A2* (Bol018458: -4.5243; Bol040704: -1.6142), and *CHS* (Bol043396: -2.2360; Bol034259:-1.3776). Expression levels of *DRL1* (Bol016365), *LAP5 (*Bol034656), *CYP98A8* (Bol039941), *ACOS5* (Bol029711) and *CYP703A2* (Bol018458) were verified by qRT-PCR with log2(2^-ΔΔCt^) values of -2.5752, -3.9456, -7.5928, -1.8604, -6.1178, -11.6789, respectively.

All 15 DEGs involved in carotenoid biosynthesis were down-regulated in the CMS line except 3 genes. Key genes were analyzed including *GELPs* (GDSL-like Lipases, Bol026926:-11.0867; Bol023605:-10.7184; Bol002897:-10.6968; Bol002899:-9.1983; Bol029971:-2.4724 and Bol026928:-10.9374), *EXL4* (extracellular lipase 4, Bol039273:-14.7554 and Bol037608:-14.7206), *CYP97A3* (Bol005984:-1.6580) and *ATA27* (Glycosyl hydrolase superfamily protein, Bol039271:-10.9311). Among them, low expression was observed in the two *EXL4*, six *GELPs* (except gene Bol029971) and *ATA27* in R2P2CMS, yet expression was high in R2P2. Log2(2^-ΔΔCt^) values of qRT-PCR analysis of two *GELPs* genes (Bol023605 and Bol002899) were -10.9400 and -10.7869, respectively.

Twelve DEGs identified as being involved in cutin, suberine and wax biosynthesis were down-regulated in R2P2CMS and key related genes were analyzed. Two *ms2/jojoba FAR2* genes (Bol010336:-1.6689; Bol007277:-1.6803) were annotated in pollen wall assembly using GO analysis. In R2P2CMS, three *CYP86C* (Bol032609: -6.4276; Bol004019: -7.1397; Bol029152:-12.8476) and four HXXXD-type acyl-transferase family proteins (Bol033609: -7.6484; Bol033614:-13.5615; Bol033616:-14.2310; Bol033612:-10.4104) were inhibited, yet highly expressed in R2P2. Furthermore, key genes *CYP704B1* (Bol023932:-1.8321) and GMC oxidoreductase (Bol029117: -4.0004; Bol045261:-2.7731) are identified.

There were 71 DEGs identified as involved in protein processing in ER and most of the up-regulated genes in the CMS line were heat shock proteins (HSPs); involved in response to stress. HSPs in the ER are thought to prevent tapetum differentiation.

### Secretion and translocation of sporopollenin precursors

Secretion and translocation of sporopollenin precursors to the microspores should be inhibited in the CMS lines. ABC (ATP binding cassette) transporters and LTPs (lipid transfer proteins) have been shown to play essential roles in secretion of sporopollenin precursors from tapetal cells and in delivery of these precursors. Here, two of the four identified ABC-2 type transporter family proteins (Bol042334: -5.7642; Bol013065: -4.9162) and three, *LTP12* (Bol004980: -11.8782; Bol010612: -17.1135; Bol014756: -10.9286) (S2 Table), showed very low expression in R2P2CMS compared to R2P2. For Bol013065, log2Ratio by RNA-seq was -4.9162 and log2(2^-ΔΔCt^) by qRT-PCR was -5.6584, which is consistent.

In addition to producing precursors for sporopollenin development, tapetum also synthesizes and secretes callose synthase (CALS), also termed β-1,3-glucanase, to degrade the callose wall surrounding the microspores after the tetrad stage. Furthermore, it is thought that the callose wall may play an important role in pollen development as a glucose source or as a stress factor forming tectum of the sexine [40]. Here, the expression level of the DEG *CALS5* (Bol019328) was extremely low in R2P2CMS, and a log2Ratio by RNA-seq of -6.1683 and log2(2^-ΔΔCt^) by qRT-PCR of -4.0327. A series of tapetum specific genes expressed at the tetrad stage are listed in Table 3. Tapetum specific proteins with unknown function, anther 20, lysine histidine transporter 2, thaumatin-like protein 3 and pectin lyase-like superfamily proteins, were all down-regulated in R2P2CMS. Expression levels of ATLP-3 (Bol039345), TAP35/TAP44 (Bol011923) and ATA20 (Bol011894) were verified with the log2(2^-ΔΔCt^) of -3.3718, -3.97709 and -14.6351.

**Table 3:** Tapetum specific genes expressed at the tetrad stage.

description		GeneID	geneLength	Means-R2P2	Means-R2P2CMS	log2Ratio(R2P2CMS/R2P2)	Probability
thaumatin-like protein 3	AT1G75030	Bol039345	744	161.21	12.78	-3.6570	0.8922
Tapetum specific protein TAP35/TAP44	AT4G20420	Bol028349	405	285.21	36.71	-2.9580	0.8877
		Bol024335	360	348.70	55.67	-2.6470	0.8838
		Bol011923	402	99.32	4.59	-4.4354	0.9017
	AT5G44540	Bol006369	354	547.98	96.41	-2.5069	0.8817
		Bol009474	363	185.97	18.34	-3.3424	0.8899
Pectin lyase-like superfamily protein	AT5G48140	Bol043084	1149	52.91	0.01	-12.3692	0.9776
		Bol033112	1194	131.01	0.05	-11.3556	0.9931
		Bol033115	1194	131.03	0.05	-11.3556	0.9931
ATA20, anther 20	AT3G15400	Bol011894	1683	4833.40	1.62	-11.5473	0.9999
		Bol011154	1866	693.08	3.88	-7.4827	0.9874
LHT2, lysine histidine transporter 2	AT1G24400	Bol041752	1326	16.13	0.6	-4.7482	0.8311
		Bol006156	1326	83.56	2.05	-5.3491	0.9216
CALS5, callose synthase 5	AT2G13680	Bol019328	5208	13.31	0.19	-6.1683	0.8519

### Proteomic differences in R2P2CMS and R2P2 by ITRAQ analysis

#### Quantification repeat analysis

**Fig 3:**
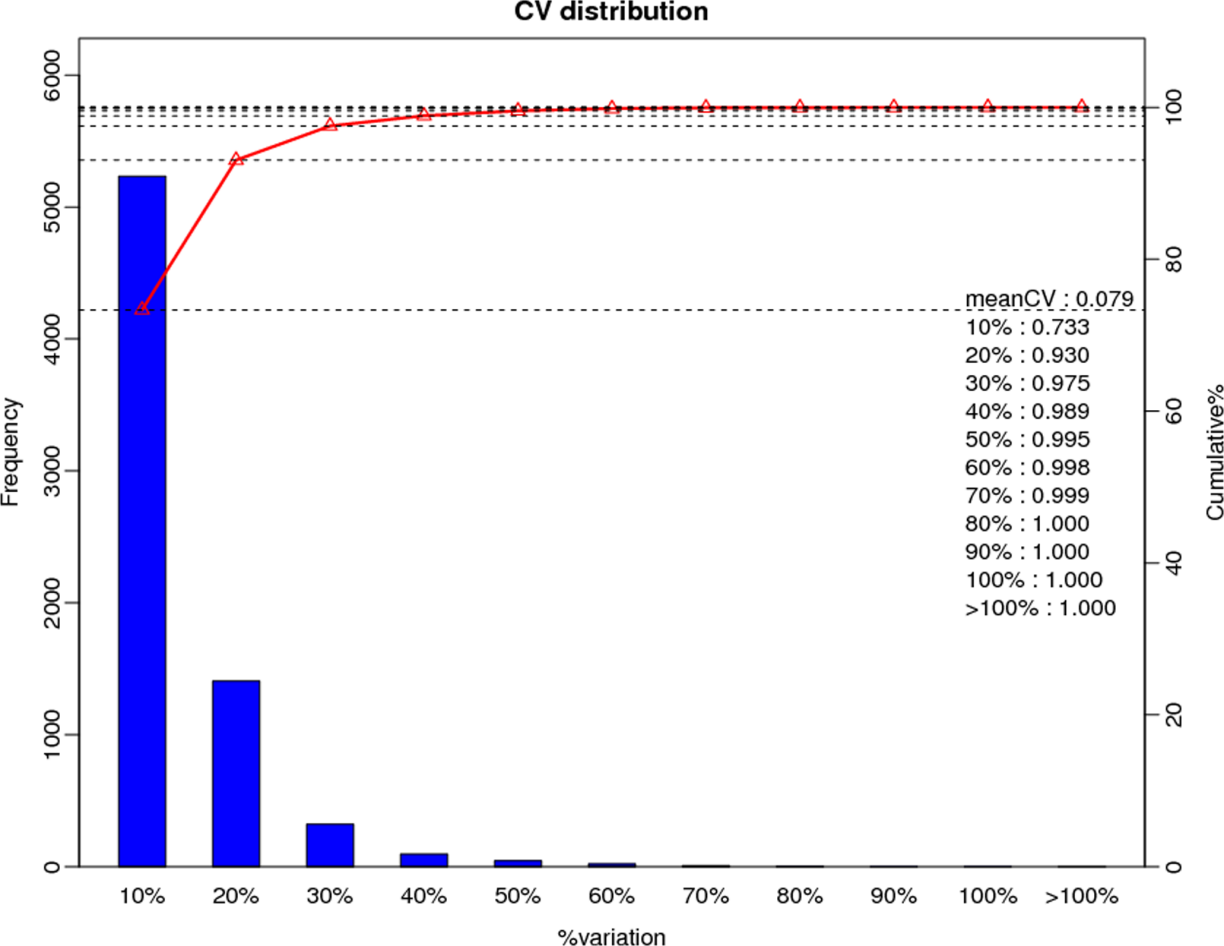
CV distribution of replicates. X-axis is the deviation between the protein ratio of the replicate samples. Y-axis is the percentage that a protein at a certain angle comprise quantified protein amount.

Here, CV was used to evaluate reproducibility. CV is defined as the ratio of the standard deviation (SD) to the mean. The lower the CV, the better the reproducibility. The mean CV for this experiment was 0.079 (Fig 3), indicating our data was reliable for further study.

In total, 274,364 spectrum were generated; 22,634 peptides and 7,147 unique proteins were identified with the cutoff: MascotPercolator Q-value ≤ 0.01. While, 833 proteins were differentially expressed including 538 up- and 295 down-regulated in R2P2CMS compared to R2P2, with a significant threshold of fold change ≥ 1.2 or ≤1/1.2 (0.8333333) and Q-value ≤ 0.05.

Among the 833 DEPs, 553 (66.39%) were classified into KEGG pathways, 6 of which were characterized as enrichment pathways with p-value < 0.05. These included ribosome (63, 11.39%), spliceome (36, 6.51%), mRNA surveillance pathway (30, 5.42%), other types of O-glycan biosynthesis(2, 0.36%), glycosphingolipid biosynthesis - ganglio series(4, 0.72%) and other glycan degradation (10, 1.81%) as shown in Table 4, which showed that substantial proteins change and glycan degradation in R2P2CMS compared to R2P2. Sporopollenin biosynthesis related pathways among KEGG analysis of DEPs include protein processing in ER (19, 3.44%), carotenoid biosynthesis (10, 1.81%), phenylpropanoid biosynthesis (17, 3.07%), flavonoid biosynthesis (7, 1.27%), biosynthesis of unsaturated fatty acids(4, 0.72%), cutin, suberine and wax biosynthesis (3, 0.54%), pathways and related DEPs were listed in S10 Table. And DEPs like cysteine proteins(Bol041861; Bol037199) and ABC transporters(Bol013065) were all up-regulated in R2P2. Lipid-transfer protein(Bol033811) was down-regulated in R2P2CMS. Key DEPs involved in pathway of protein processing in ER, except for Bol013102, all the HSPs (Bol039505, Bol036095, Bol032900, Bol039198, Bol010195) were up-regulated in the CMS line, which was consistent with the results of transcriptome. In carotenoid biosynthesis pathway, 5 GDSL-like Lipases(Bol026926, Bol035147, Bol026928, Bol035145, Bol002897) and 2 DEPs EXL4 (Bol037608, Bol039273)were all up-regulated in CMS line. Key DEPs CYP98A8 Bol039941) in phenylpropanoid biosynthesis was up-regulated in R2P2CMS, other key DEPs like DRL1, LAP5, LAP6, ACOS5, and PAL1 found in DEGs were not identified. In flavonoid biosynthesis, key DEPs participated include CYP98A8(Bol039941,+), CHS (Bol043396,-) CYP82F1 (Bol033613, -).

**Table 4:** KEGG analysis of DEPs.

Pathway	DEPs with pathway annotation (553)	P-value	Pathway ID
Ribosome	63 (11.39%)	1.024331e-14	ko03010
mRNA surveillance pathway	30 (5.42%)	4.796534e-05	ko03015
Spliceosome	36 (6.51%)	0.0007663486	ko03040
Other types of O-glycan biosynthesis	2 (0.36%)	0.03342961	ko00514
Glycosphingolipid biosynthesis - ganglio series	4 (0.72%)	0.03460699	ko00604
Other glycan degradation	10 (1.81%)	0.03708956	ko00511

### Correlation analysis of DEGs and DEPs

**Fig 4:**
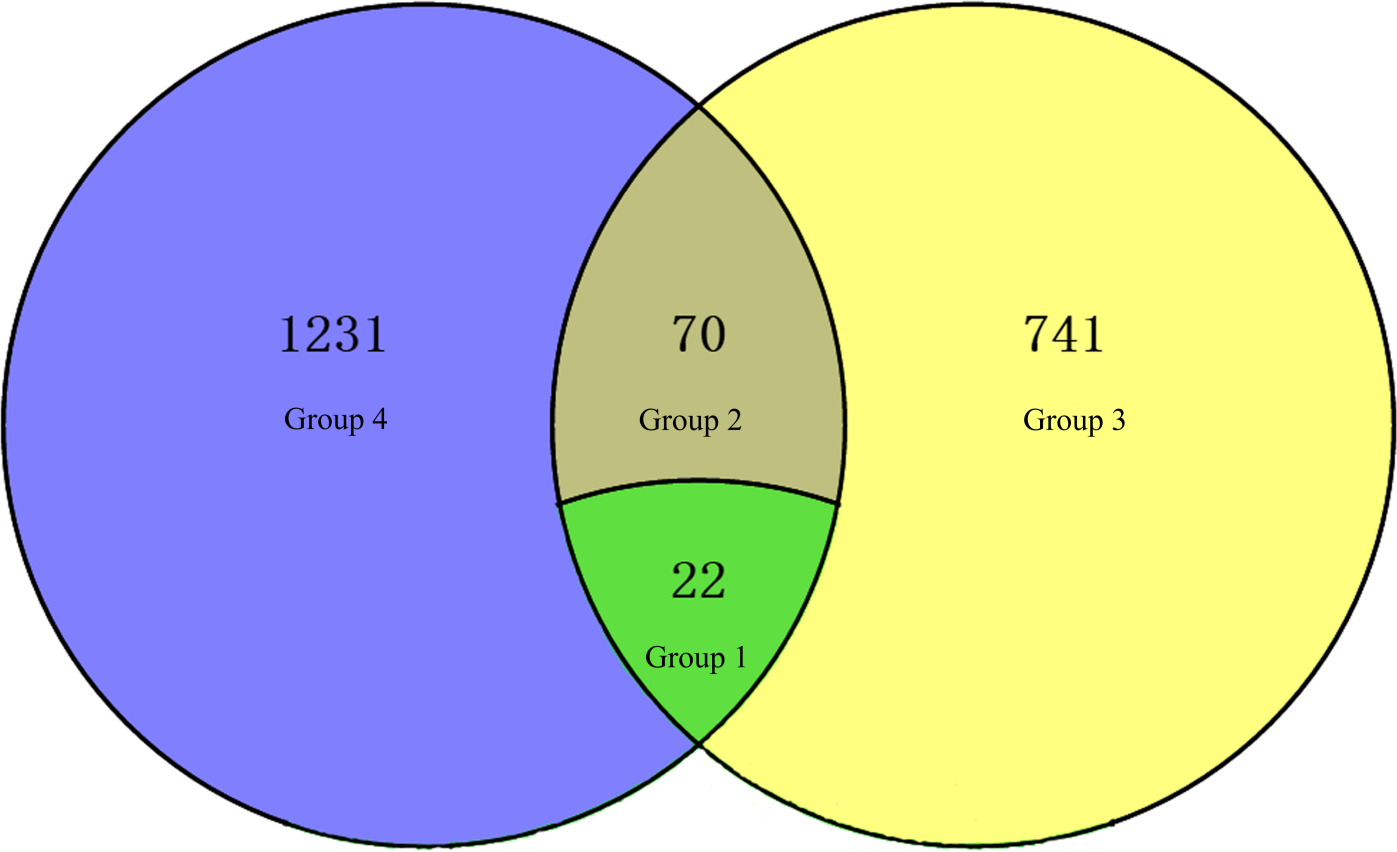
Venn diagram of numbers of DEGs and DEPs.

Correlation analysis of DEGs and DEPs was performed between R2P2CMS and R2P2. DEGs and DEPs were divided into four groups according to expression in R2P2CMS compared to R2P2: Group 1, 22 DEGs and DEPs (13 down-regulated and 9 up-regulated) with the same expression; Group 2, 70 DEGs and DEPs with opposite expression; Group 3, 741 DEPs whose corresponding genes were not differentially expressed; Group 4, 1232 DEGs whose corresponding proteins were not differentially expressed. DEGs and DEPs are displayed in the Venn diagram (Fig 4).

### KEGG pathways analysis of all the correlated DEGs/DEPs

**Fig 5:**
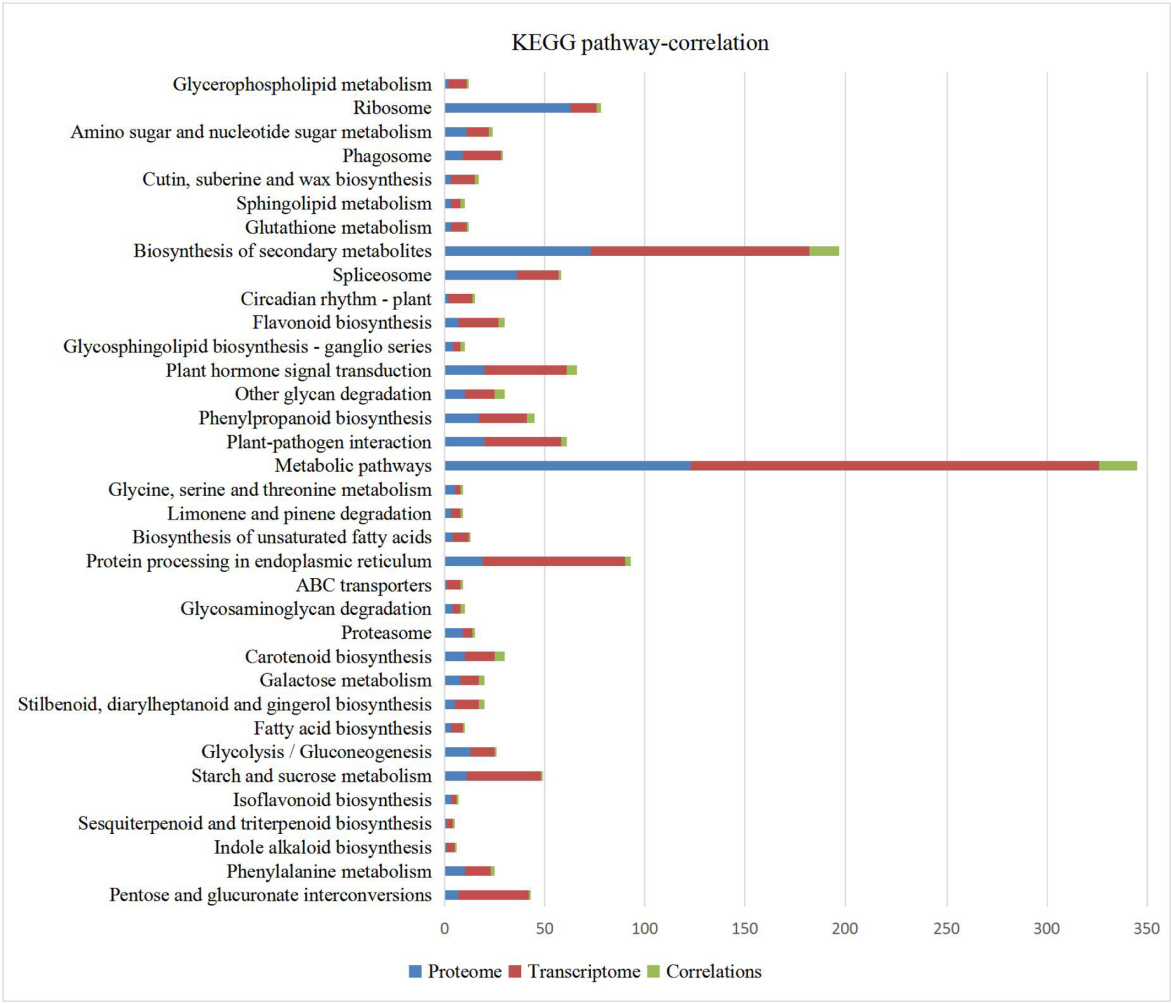
KEGG pathways of DEGs and DEPs. X-axis represents the numbers of DEGs or DEPs.

KEGG pathway analysis of all 92 DEGs/DEPs are shown in Fig 5. Among them, pathways identified include: phenylalanine metabolism (Bol005311, Bol013614), isoflavonoid biosynthesis (Bol041944), fatty acid biosynthesis (Bol008037), ABC transporters (Bol013065), cutin, suberine and wax biosynthesis (Bol032609, Bol004019) and protein processing in ER (HSPS: Bol013102, Bol039505, Bol039198). Except for Bol013102, the expression pattern of other two HSPs in was the same. Key CHS(Bol043396) in flavonoid biosynthesis pathway in both analysis was all down-regulated, while CYP98A8 Bol039941) had the opposite expression pattern. Only one cysteine protein Bol041861 was correlate, but the expression pattern was opposite. 2 DEPs EXL4 (Bol037608, Bol039273) and 3 GDSL-like Lipases Bol026926, Bol002897 and Bol026928 with opposite expression pattern were all correlated in carotenoid biosynthesis pathway. In phenylpropanoid biosynthesis other key genes like DRL1, LAP5, LAP6, ACOS5, and PAL1 found in DEGs were all not identified, only DEPs CYP98A8 Bol039941) up-regulated in R2P2CMS was correlate and it’s expression pattern was opposite. ABC transporters (Bol013065) in both analysis was opposite. In biosynthesis of unsaturated fatty acids, only one NAD(P)-binding Rossmann-fold superfamily protein Bol008037 was correlated with opposite expression pattern. The glycan degradation pathway was identified in both transcriptome and proteome analysis including GELPs (Bol002897 and Bol026928), BGAL (beta-galactosidase, Bol035797 and Bol000448) and EXL6 (extracellular lipase 6, Bol039272) and were expressed at a very low level in R2P2CMS. GELPs (Bol002897 and Bol026928) are also involved in the carotenoid biosynthesis pathway, and EXL6 may play an essential role in pollen development in B. rapa and Arabidopsis [41]. Among DEGs and DEPs with the same trend pattern, EF hand calcium-binding protein family(Bol026249, -3.8032; Bol015339, -3.8569), embryo-specific protein 3 (ATS3, Bol020675, AT5G62210–3.5356) which was participated in reproduction developmental process by GO annotation, late embryogenesis abundant domain-containing protein(Bol042711, AT2G03740–16.9392) which was verified to be accumulated late in embryogenesis of cotton[42], lipid-transfer protein(Bol033811, -11.5318), CHS Bol043396, -2.2360) may be important for resulting in the sterility in R2P2CMS.

## Discussion

### Tapetum PCD is delayed in R2P2CMS

Because of the low correlation of transcriptome and proteome analysis, the following discussion mainly focus on transcriptome analysis. Delayed degradation of tapetum was responsible for pollen dysfunction. The signal for tapetum degradation by PCD commenced at the tetrad stage [43]. Previous studies on OguCMS showed that abnormal activity of tapetum appeared at anther developmental stages 7–10 [22,44]. Our cytological analysis revealed proliferation and expansion of tapetum at anther developmental stage 7–10 and most of the microspores were compressed by abnormal hypertrophic tapetum cells in R2P2CMS. Thickening tapetum instead of vacuolization and degradation of the tapetum at the tetrad stage (7–8) and microspore stage (9–10) in R2P2CMS is likely to be the cause of sterility. The expression levels of DEGs related to tapetum PCD were all significantly down-regulated in R2P2CMS compared to R2P2. A GA-regulated family protein, *MYB101* involved in the GA mediated signaling pathway triggered PCD, *TDF1/MYB 35*, *PLP4*, *AMS* and the cysteine proteinase superfamily. The cytological analysis and corresponding transcriptome data verified that the sterility of R2P2CMS may be due to delayed degradation of tapetum.

### Sporopollenin biosynthesis related pathways and key genes are inhibited in R2P2CMS

Sporopollenin is the main constituent of exine which is composed of the outer sexine (tectum, bacula tryphine) and the inner nexine. Tapetal cells are essential for pollen development and secrete precursors via a catalytic reaction into the exine. Furthermore, the degradation products of tapetum are precursors for sporopollenin and tryphine biosynthesis at the tetrad stage [35]. Brassicaceae have unique secretory tapetum, which means sporopollenin precursors, proteins and lipids produced through tapetum PCD are part of the pollen exine. Studies showed that the important components of sporopollenin are fatty acids and phenylpropanoids and the components of tryphine (pollen coat) contain flavonoids and carotenoids [45,46]. Our pathway enrichment analysis showed that sporopollenin biosynthesis related pathways like fatty acid elongation, biosynthesis of unsaturated fatty acid, phenylpropanoid biosynthesis pathway, flavonoid biosynthesis and carotenoid biosynthesis were among the top 20 significantly enriched pathways. Sporopollenin and waxes or cutin biosynthesis pathways share components [47,48]; the cutin, suberine and wax biosynthesis pathways were also identified herein. Fatty acid modifications like unsaturation and elongation take place in the tapetum ER [47] and changes along with degradation of tapetum include dilation of cisternae in ER and fusion of stacked ER into the plasma membrane [46,49]. The pathway of ER protein processing was also included in the top 20 pathways.

Key genes involved in sporopollenin biosynthesis were analyzed here to elucidate the relationship between expression and sterility. In the *Arabidopsis male sterility 2(ms2)-*defective mutant, an absent pollen exine layer was observed [50]. In our study, two *ms2/jojoba FAR2* genes (Bol010336, Bol007277), involved in cutin, suberine and wax biosynthesis pathways, were significantly down-regulated in R2P2CMS. Our KEGG analysis showed that biosynthesis of unsaturated fatty acid and fatty acid elongation may be involved in cutin, suberine and wax biosynthesis pathways. *Ms2*/*jojoba FAR2* genes were confirmed *in vitro* to convert VLCFAs (very long-chain fatty acids) into alcohols, which are critical for sporopollenin biosynthesis [45,51]. During cutin, suberine and wax biosynthesis, FAR converts a long-chain acyl-CoA into a long-chain primary alcohol. Glucose-methanol-choline (GMC) oxidoreductase family proteins were shown to regulate tapetum degeneration and pollen exine formation and were specifically expressed in tapetal cells and microspores [52]. Furthermore, GMC was proposed to catalyze α-,ω-dicarboxylic FAs involved in the development of surface cutin [53]. Here, 2 GMC oxidoreductase (Bol029117, Bol045261) involved in cutin, suberine and wax biosynthesis were down-regulated in R2P2CMS, and oxidate ω-hydroxy FA into ω-acid oxo FA function downstream at CYP86. Two other GMC oxidoreductases, Bol034841 and Bol026297, were identified with the log2Ratio (R2P2CMS/R2P2) of -5.2882 and -4.4747, respectively. Acyl-CoA synthetase 5 (*ACOS5)* is thought to play a key role in exine formation in *Arabidopsis* [54]. In addition, *OsACOS12*, similar to A*COS*5 in rice [55], was found to function in pollen wall formation and tapetum PCD and the defective mutant displayed male sterility. Here *ACOS5* (Bol029711), involved in phenylpropanoid biosynthesis and pollen exine formation, was down-regulated in R2P2CMS. Another key enzyme in the phenylpropanoid biosynthesis, PAL1 (phenylalanine ammonia lyase 1, Bol037689 and Bol025522), was also significantly down-regulated in R2P2CMS and participated in gametophyte development annotated by GO. PAL1 converts phenylalanine to cinnamic acid and functions upstream of this pathway. Cinnamic acid is then converted to CoA-type compounds by ACOS5, and converted to aldehydes by NAD(P)-binding Rossmann-fold superfamily protein and DRL1 (dihydroflavonol 4-reductase-like 1). Finally lignin is produced by POD. Here five NAD(P)-binding Rossmann-fold superfamily protein genes (Bol024018, Bol011801, Bol016365, Bol005963, Bol027627), one DRL1 gene (Bol016365) and four POD genes (Bol020252, Bol025982, Bol013614 and Bol040965) were expressed at a very low level in R2P2CMS. Cytochrome P450s are necessary for exine formation, like *CYP703A2* [56] and *CYP704B1* [57] in *Arabidopsis*, and *CYP704B2* in rice [48]. Our results showed that *CYP703A2* (Bol018458, Bol040704), which is involved in flavonoid biosynthesis, and *CYP704B1* (Bol023932) and *CYP86* (Bol032609, Bol004019, Bol029152), which are involved in cutin, suberine and wax biosynthesis for pollen exine formation, were significantly down-regulated in the CMS line. In addition, *CYP78A7* (Bol043713) and *CYP98A8* (Bol039941), both involved in flavonoid and phenylpropanoid biosynthesis, were also down-regulated in R2P2CMS. Chalcone synthase (CHS), a key enzyme involved in flavonoid biosynthesis, was predominantly or specifically expressed in the tapetum [58,59]. In a wheat and triticale study [60], *TaCHSL1A* (chalcone synthase-like gene) was detected only in the tapetum during the "free" and early vacuolated microspore stages. In *Raphanus sativus* with OguCMS [14], *CHS* was uniquely suppressed in the CMS lines and *CHS-*dependent flavonols were required for the development of functional pollen. In this study, 2 *CHS* genes (Bol043396, Bol034259) were inhibited in R2P2CMS. Loss of LAP5 and LAP6-CHS family protein double mutant produced pollen without exine and showed strong male sterility [61]. *LAP5* (Bol013698, Bol034656) and *LAP6* (Bol025267), involved in flavonoid biosynthesis and phenylpropanoid metabolic process for pollen wall assembly by GO process analysis, were down-regulated in R2P2CMS. Studies showed that *LAP3* and *DRL1* mutants produced abnormal pollen [62–64]. Here, *LAP3* (Bol037639, Bol045568) genes, involved in pollen wall assembly, and *DRL1* (Bol016365), involved in flavonoid biosynthesis and phenylpropanoid biosynthesis for pollen exine formation, were all down-regulated in R2P2CMS. *CHS* and *LAP5* or *LAP6* function upstream of flavonoid biosynthesis to convert cinnamoyl-CoA to chalcones. Ten percent of BrGELPs were expressed in fertile buds but not sterile buds in *B*. *rapa* [41]. In the carotenoid biosynthesis pathway, we identified 6 GELPs (GDSL-like Lipases, Bol026926, Bol023605, Bol002897, Bol002899, Bol029971 and Bol026928), *CYP97A3* (Bol005984), *ATA27* (Glycosyl hydrolase superfamily protein, Bol039271), an anther-specific gene in *Arabidopsis* [65] and two genes *EXL4* (extracellular lipase 4, Bol039273 and Bol037608) required for efficient hydration of *Arabidopsis* pollen [66], which were all inhibited in R2P2CMS.

### Secretion and translocation of sporopollenin are inhibited in R2P2CMS

In *Arabidopsis*, ABC transporters play an essential role in cuticular lipid secretion in wax and cutin [67,68]. It is interesting that some of the ABC transporters are expressed specifically in tapetum in *Arabidopsis* and rice [69,70]. We hypothesize that ABC transporters may participate in the secretion of sporopollenin precursors from tapetal cells. Here, we identified four ABC-2 type transporter family proteins which were down-regulated in R2P2CMS. The characteristics of LTPs make them candidates for translocation of the sporopollenin precursors from tapetum to microspores in Brassicaceae [47]. In the tapetum, antisense down-regulation of tomato *GRP92*, a homologous gene of LTPs, resulted in an abnormal exine [71]. Three *LTP12* DEGs were expressed at very low levels in R2P2CMS compared to R2P2, and were in the cell wall responsible for establishment of localization by GO Process. *LTP12* may play a role in delivery of these precursors.

### Low correlation between transcriptome and proteome data

Only a small number of DEGs and DEPs were correlated. Inconsistency between transcriptome and proteome data have also been reported in soybean [72] and citrus [73]. (1) the two biological processes are non-equivalence in nature; (2) the quantity and quality of proteomics data are largely affected by their modification and degradation; (3) the greater numbers of DEGs may due to the more mature RNA-Seq technology than iTRAQ technology.

## Conclusion

**Fig 6:**
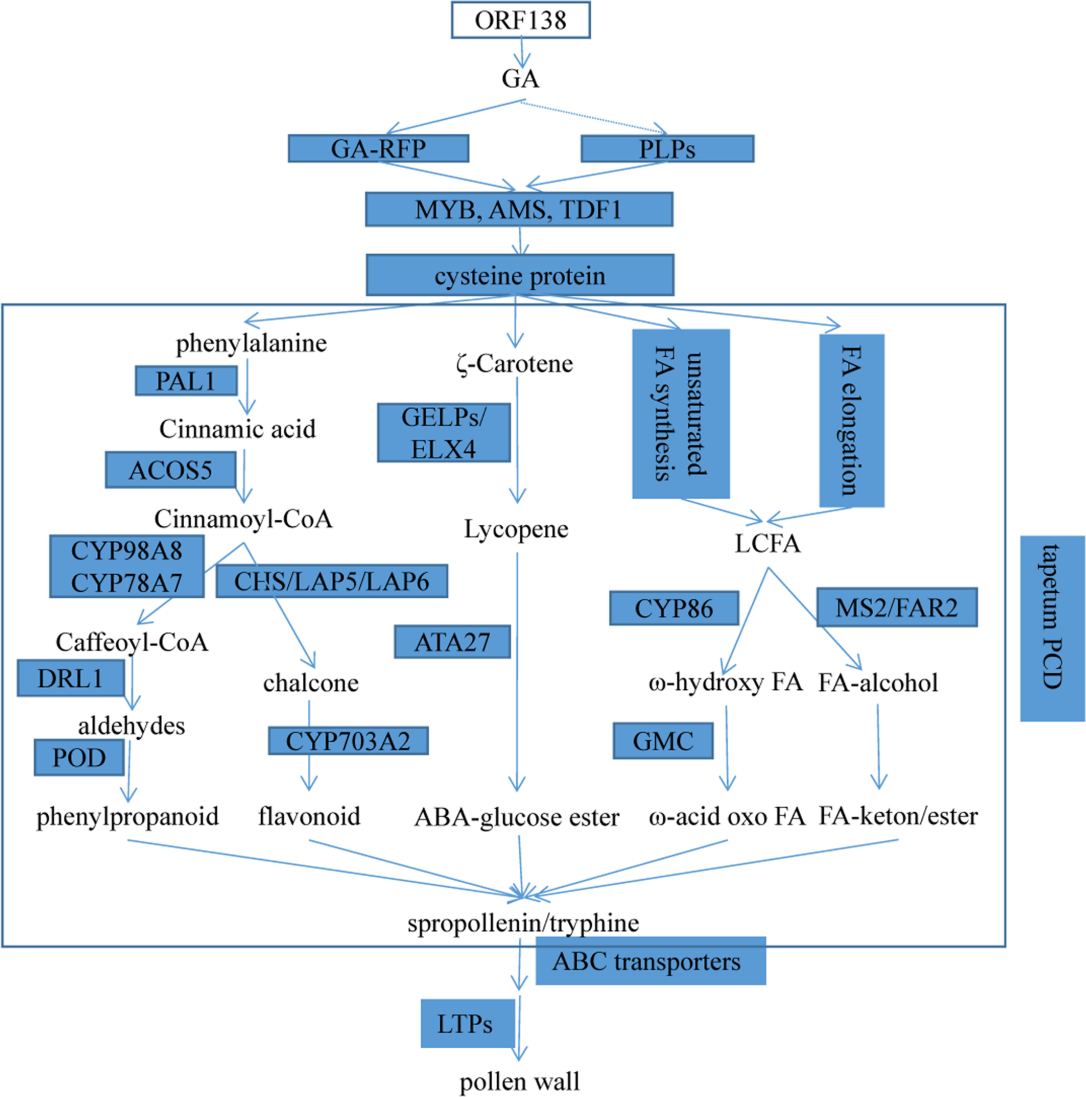
Pollen wall development and key genes in cabbage. GA-RFP: GA-regulated family protein. LCFA: long-chain fatty acid. LTPs: lipid transfer proteins.

By combining transcriptome and proteome, we identified key genes and pathways which may participate in tapetum PCD and sporopollenin synthesis for pollen wall development in cabbage. Tapetum PCD at the tetrad stage is regulated by the GA mediated signaling pathway, in which DEGs such as GA-regulated family proteins, *PLP4*, and transcription factors like TDF1, bHLH type-AMS, MYB were suppressed in R2P2CMS. Cysteine proteases function downstream to regulate tapetal degeneration. Sporopollenin, the main constituent of exine, is synthesized through different metabolic pathways: phenylpropanoid biosynthesis pathways, flavonoid biosynthesis, carotenoid biosynthesis, biosynthesis of unsaturated fatty acid, and fatty acid elongation. The key genes participating in these pathways such as *PAL1*, *ACOS5*, *DRL1*, *CYP*, *POD*, *CHS*, *LAP5*, *LAP6*, *GELP*, *ELX4*, *ATA27*, *GMC*, *MS2/FAR2* were all significantly down-regulated in R2P2CMS, that’s why no pollen in the CMS line. In phenylpropanoid and flavonoid biosynthesis, *PAL1* and *ACOS5* are the key upstream genes, by them phenylalanine is transformed into cinnamoyl-CoA. Then under the functions of *CYP*, *DRL1* and *POD*, cinnamoyl-CoA is finally transformed into phenylpropanoid, and in another pathway, through *DRL1*, *LAP5*, *LAP6* and *CYP*, flavonoid is synthesized. In carotenoid biosynthesis, key genes *GELP* and *EXL4* transform ζ-Carotene into lycopene, then under the function of *ATA27*, ABA-glucose ester is synthesized. In unsaturated fatty acid and fatty acid elongation pathway, long-chain fatty acid(LCFA) is converted into FA-alcohol by MS2/FAR2, which are critical for sporopollenin biosynthesis. After the function of CYP and GMC were shown to regulate tapetum degeneration and pollen exine formation, LCFA is converted into ω-acid oxo FA function downstream at CYP86. After sporopollenin synthesis, secretion and translocation of sporopollenin precursors takes place regulated by ABC transporters and lipid transfer proteins. Cytological analysis showed that hypertrophic abnormal tapetum rather than degradation at the tetrad stage resulted in the delayed deposition of sporopollenin responsible for the sterility of R2P2CMS. Based on these results, a schematic diagram (Fig 6) was constructed which provides a comprehensive understanding of the mechanism underlying OguCMS in cabbage.

## Supporting information

S1 Table Primers and results of qRT-PCR for selected genes. (XLSX)

S2 Table Genes related to tapetum degeneration through PCD. (XLS)

S3 Table Genes involved in fatty acid elongation. (XLSX)

S4 Table Genes involved in biosynthesis of unsaturated fatty acid. (XLS)

S5 Table Genes involved in phenylpropanoid biosynthesis pathways. (XLSX)

S6 Table Genes involved in flavonoid biosynthesis. (XLSX)

S7 Table Genes involved in carotenoid biosynthesis. (XLS)

S8 Table Genes involved in cutin, suberine and wax biosynthesis. (XLS)

S9 Table Genes involved in protein processing in the endoplasmic reticulum. (XLS)

S10 Table DEPs involved in sporopollenine and pollen wall development. (XLSX)
